# The first two complete mitochondrial genomes for the genus *Neotrichoporoides* (Hymenoptera, Eulophidae) and their phylogenetic analysis

**DOI:** 10.3897/zookeys.1291.206033

**Published:** 2026-09-04

**Authors:** Jun-Sheng Wen, Xiang-Xiang Jin, Wen-Jian Li, Zhi-Peng Chen, Guo-Hao Zu

**Affiliations:** 1 College of Horticulture and Landscape, Tianjin Agricultural University, Tianjin 300392, China College of Horticulture and Landscape, Tianjin Agricultural University Tianjin China; 2 Guangdong Key Laboratory of Animal Conservation and Resource Utilization, Institute of Zoology, Guangdong Academy of Sciences, Guangzhou 510260, China Guangdong Key Laboratory of Animal Conservation and Resource Utilization, Institute of Zoology, Guangdong Academy of Sciences Guangzhou China; 3 Jiangsu Provincial Key Laboratory of Coastal Wetland Bioresources and Environmental Protection, School of Wetland, Yancheng Teachers University, Yancheng 224007, China Jiangsu Provincial Key Laboratory of Coastal Wetland Bioresources and Environmental Protection, School of Wetland, Yancheng Teachers University Yancheng China

**Keywords:** Eulophidae, gene rearrangement, mitochondrial genome, mitogenomic diversity, parasitic wasps, phylogeny, selection pressure

## Abstract

*Neotrichoporoides* belongs to the family Eulophidae (Hymenoptera: Chalcidoidea). As a group of parasitic wasps, it plays an indispensable role in the biological control of agricultural and forest pests and in maintaining ecosystem balance. To date, only nine complete mitochondrial genomes of Eulophidae have been sequenced worldwide, including the two newly sequenced species in this study. To enrich our understanding of the mitochondrial genomic diversity of Eulophidae and to provide preliminary insights into its phylogenetic relationships, we sequenced and comparatively analyzed the mitochondrial genomes of two *Neotrichoporoides* species. The mitogenomes of *N. nyemitawus* (GenBank: PZ188956; 15,164 bp) and *N. viridimaculatus* (GenBank: PX794932; 15,297 bp) contain 13 protein-coding genes (PCGs), 22 transfer RNAs (tRNAs), two ribosomal RNAs (rRNAs), and one control region (CR), and exhibit a strong AT bias, with AT contents of 85.5% and 85.0%, respectively. We further analyzed mitochondrial gene rearrangements across 17 species from Encyrtidae, Eulophidae and Pteromalidae and summarized family-specific rearrangement characteristics. tRNA rearrangements were detected in all three families. Eulophidae harbors conserved PCGs, while the inverse transposition of *trnA* and transposition of *trnV* are likely reported for the first time within this family. The two *Neotrichoporoides* species differ only in the arrangement of several tRNAs. Comparative analysis of PCGs revealed differences in molecular evolutionary rates among genes, with *ATP8*, *ND2* and *ND4* evolving faster than the others. Phylogenetic analysis based on mitochondrial genome sequences showed that species from two subfamilies formed a monophyletic group, and congeneric species clustered into a single clade. This study contributes to resolving phylogenetic relationships within Eulophidae and further deepens our understanding of this family.

## Introduction

The family Eulophidae is one of the most species-rich and ecologically critical groups within the superfamily Chalcidoidea, encompassing diverse life strategies including parasitoids and gall inducers, and plays an irreplaceable role in the biological control of agricultural and forestry pests as well as in maintaining ecosystem balance (Gauthier et al. 2000; Mendel et al. 2004). According to the authoritative website UCD Community (2023), the Eulophidae currently comprises 328 genera and more than 6000 described species worldwide (UCD Community 2023).

The genus *Neotrichoporoides* (Chalcidoidea: Eulophidae) was established based on its type species, *Neotrichoporoides uniguttatus*Girault (1913). Over nearly a century of taxonomic revisions, eight generic names have been synonymized under it, including *Aprostoceroloides*, *Tetrastichomorpha*, *Trichaporoidella*, *Epiquadrastichus*, *Paraprostocetus*, *Burksia*, *Neogaleopsomyia* and *Dubiostalon*. The circumscription of the genus was clarified by Graham (1987) and Bouček (1988), with subsequent minor additions by Narendran (2007) and Burks (2012). Currently, the genus is widely accepted within the subfamily Tetrastichinae of Eulophidae and comprises approximately 75 valid species (UCD Community 2023). Li and Li (2021) described two new species of *Neotrichoporoides* from China, which exhibit the diagnostic characters of the genus, including body color usually dark with distinct metallic luster, or entirely yellow and dull; female antenna with four disc-shaped ring segments (annelli); and a propodeum much longer than the postscutellum, with strong reticulate sculpture.

Insect mitochondrial genomes are small circular molecules containing 37 genes and a control region. Owing to their conserved gene content and arrangement, maternal inheritance, and relatively rapid evolutionary rate, they have become important molecular markers (Boore 1999; Krzywinski et al. 2006). Rasplus et al. (2020) confirmed the monophyly of Eulophidae based on phylogenomic data, and Cruaud et al. (2024) subsequently placed it within the “small wasp clade” at the base of Chalcidoidea, further clarifying its phylogenetic relationship with Trichogrammatidae. In addition, wing interference patterns (WIPs), when combined with molecular data, have shown promising application in the identification of cryptic species (Shevtsova et al. 2011). These studies collectively indicate that an integrative taxonomic approach is essential for understanding the evolutionary history of Eulophidae. Although research approaches have continuously evolved from morphological traits to molecular markers and genomics, and phylogenetic studies of Eulophidae have progressed considerably, generic relationships have long been difficult to resolve reliably using morphology and traditional molecular markers alone (Rasplus et al. 2020). In contrast, mitochondrial genomes, characterized by conserved gene content and stable gene arrangement, exhibit a sequence evolutionary rate that is faster than that of nuclear genes—a feature that renders them particularly valuable for phylogenetic studies of closely related taxa and for research on adaptive evolution (Boore 1999).

In this study, we sequenced and annotated the mitochondrial genomes of *Neotrichoporoides nyemitawus* Rohwer, 1921 (accession no. PZ188956) and *Neotrichoporoides viridimaculatus* Fullaway, 1955 (accession no. PX794932), analyzed their genomic features, and reconstructed molecular phylogenetic relationships by combining them with published data from other Eulophidae species. The newly obtained molecular data will enhance our understanding of mitogenomic characteristics in Eulophidae. Furthermore, by combining these two mitogenomes with six previously deposited eulophid mitochondrial genomes, we performed comparative genomic and phylogenetic analyses to determine the taxonomic position of the two *Neotrichoporoides* species within the mitogenomic diversity of Eulophidae.

## Materials and methods

### Sample collection, DNA extraction and sequencing

Specimens of *N. nyemitawus* were collected in August 2022 from the Tianjin Academy of Agricultural Sciences (39°5'54"N, 117°3'8"E), Xiqing District, Tianjin City, China, and specimens of *N. viridimaculatus* were collected in June 2025 from Machang Village (33°33'43"N, 118°43'33"E), Siyang County, Suqian City, Jiangsu Province, China. Freshly collected specimens were immediately immersed in 99% ethanol for initial preservation and subsequently stored in a –20 °C freezer in the Insect Herbarium of Tianjin Agricultural University. After morphological identification, total DNA was extracted from each specimen (excluding the abdomen) using the DNeasy Blood & Tissue Kit (Qiagen, Hilden, Germany) following the manufacturer’s instructions. The purity and concentration of the extracted total DNA were assessed by 1% agarose gel electrophoresis and optical density measurements. Total DNA of the two eulophid species was sequenced using the Illumina NovaSeq 6000 platform with a 350 bp insert size and a paired-end 150 bp sequencing strategy. Sequencing was performed by Novogene Co., Ltd (Beijing, China).

### Mitogenome assembly, annotation and analysis

After removing adapter sequences from the raw data, low-quality reads and adapter contamination were filtered using fastp v. 0.23.4 (Chen et al. 2018). Clean data with a Phred quality score ≥Q30 and a minimum of 4 Gb per sample were retained, providing a reliable basis for subsequent genome assembly and analysis. To improve the accuracy and completeness of mitochondrial genome assembly, two independent bioinformatics pipelines were employed: MitoZ v. 3.6 (Meng et al. 2019) and Get Organelle v. 1.7.7.0 (Jin et al. 2020). Using the mitochondrial genome sequences of related Eulophidae species published in the GenBank database as a reference sequence set (e.g., *Chouioia cunea*, *Tamarixia radiata*, *Tetrastichus howardi*), preliminary annotation was performed with the Mitos 2 online server (http://mitos2.bioinf.uni-leipzig.de) on the Galaxy online platform (The Galaxy Community 2024). Subsequently, manual curation and adjustment of gene boundaries and coding-region integrity were carried out using Geneious Prime software to ensure annotation accuracy. For tRNA secondary structure prediction and visualization, secondary structures were first predicted on the Galaxy platform and then visualized and validated using VARNA v. 3.9 (Darty et al. 2009). The circular map of the mitochondrial genome was drawn using the CGview Server online tool. Nucleotide composition, relative synonymous codon usage (RSCU), and selection pressure (Ka/Ks) were analyzed using MEGA v. 11.0.13 (Tamura et al. 2021). Nucleotide skewness was calculated using the following formulas to assess the asymmetry of genomic base composition: AT-skew = (A–T)/(A+T), GC-skew = (G–C)/(G+C) (Perna and Kocher 1995; Hassanin et al. 2005). Tandem repeats in the CR were identified by Tandem Repeats Finder (Benson 1999).

### Molecular phylogenetic analyses

For phylogenetic analysis, nine species from two families of Chalcidoidea were selected, including eight Eulophidae species and an Encyrtidae species as an outgroup (Table 1). Phylogenetic trees were reconstructed using maximum likelihood (ML) and Bayesian inference (BI). Each protein-coding gene was individually aligned using the L-INS-i strategy of the MAFFT v. 7 online service, followed by refinement with MACSE software (Ranwez et al. 2018; Katoh et al. 2019). The alignments were trimmed using GBlocks, and the trimmed sequences of all genes were concatenated into a protein-coding gene dataset using PhyloSuite v. 1.2.3 (Talavera and Castresana 2007; Zhang et al. 2020). The best-fit nucleotide substitution model was selected using ModelFinder v. 2.2.0 based on the Bayesian information criterion (BIC) (Kalyaanamoorthy et al. 2017). BI analysis was performed with MrBayes v. 3.2.7a using four Markov chains (Ronquist et al. 2012), running for 2 million generations, sampling every 1000 generations, with the first 25% of trees discarded as burn-in. Convergence was considered reached when the average standard deviation of split frequencies was below 0.01, and the potential scale reduction factor (PSRF) approached 1.0. ML analysis was conducted using IQ-TREE v. 2.2.0 (Nguyen et al. 2015) with 1000 bootstrap replicates. The resulting phylogenetic trees were then enhanced using the iTOL online tool (https://itol.embl.de).

**Table 1.** Mitochondrial genome information for the eight Eulophidae species and the outgroup species. Asterisks (^*^) indicate the two newly sequenced species in this study.

**Superfamily**	**Family**	**Species**	**Size (bp)**	**ID**
Chalcidoidea	Encyrtidae	*Exoristobia philippinensis* Ashmead, 1904	15751	NC_084171
	Eulophidae	*Aulogymnus japonicus* Ashmead, 1904	16961	PQ776751
		*Chouioia cunea* Yang, 1989	14930	NC_060368
		*Citrostichus phyllocnistoides* Narayanan, 1960	14669	PQ059861
		*Neotrichoporoides nyemitawus* Rohwer,1921^*^	15164	PZ188956
		*Neotrichoporoides viridimaculatus* Fullaway, 1955^*^	15297	PX794932
		*Tamarixia radiata* Waterston, 1922	14752	MN123622
		*Tetrastichus howardi* Olliff, 1893	14791	NC_079567
		*Tetrastichus* sp.	14810	PP735728

## Results

### Mitogenome organization and nucleotide composition

**Figure 1.**
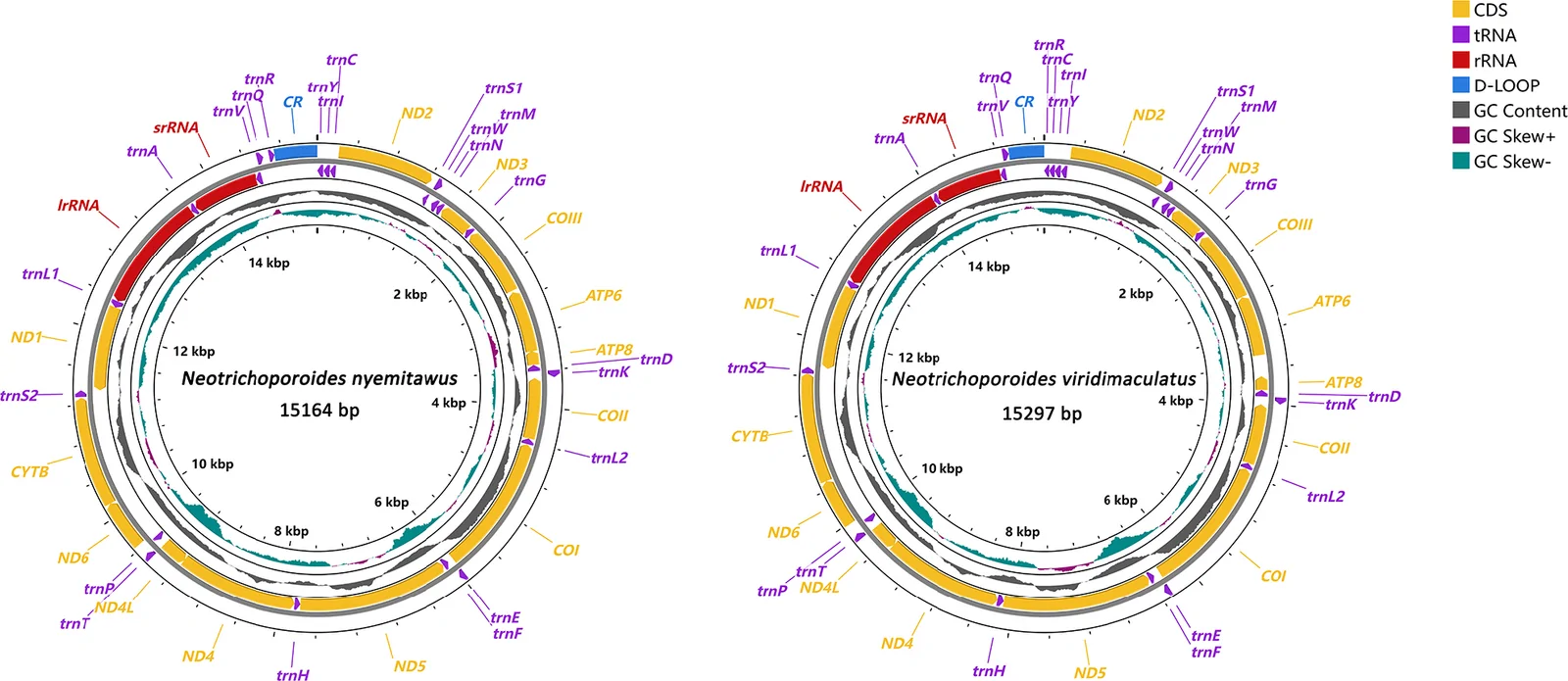
Circular map of the mitochondrial genome of *N. nyemitawus* and *N. viridimaculatus*. Genes are color-coded by functional category as indicated. Genes on the majority strand and minority strand are placed on the outer and inner sides, respectively; the second and third inner circles display GC content, GC-skew+, and GC-skew–. Gene names follow standard insect mitochondrial abbreviations.

The mitochondrial genomes of *N. nyemitawus* and *N. viridimaculatus* are 15,164 bp and 15,297 bp in length, respectively. The two species share an identical gene composition, each containing 13 protein-coding genes, 22 tRNA genes, two rRNA genes, and a control region located between *trnM* and *trnI* (Fig. 1). On the majority strand, both species encode three protein-coding genes (*ND2*, *ND6*, *CYTB*) and five tRNA genes (*trnW*, *trnK*, *trnE*, *trnT*, *trnS2*). The minority strand comprises the remaining ten protein-coding genes (*COI*, *COII*, *COIII*, *ND1*, *ND3*, *ND4*, *ND4L*, *ND5*, *ATP6*, *ATP8*), 16 tRNA genes (*trnC*, *trnY*, *trnI*, *trnS1*, *trnN*, *trnM*, *trnG*, *trnD*, *trnL2*, *trnF*, *trnH*, *trnP*, *trnL1*, *trnA*, *trnV*, *trnQ*), and the two rRNA genes (*lrRNA*, *srRNA*). However, the coding strand of *trnR* differs between the two species: it is encoded on the sense strand in *N. nyemitawus* but on the antisense strand in *N. viridimaculatus* (Table 2). Both genomes exhibit intergenic nucleotide overlaps of 1 to 7 bp in length between adjacent genes. *Neotrichoporoides nyemitawus* has 12 such overlapping regions, whereas *N. viridimaculatus* has 11. The longest overlap (7 bp) is located between *ATP6* and *ATP8* as well as between *ND4* and *ND4L* in *N. nyemitawus*, and between *ND4* and *ND4L* in *N. viridimaculatus*. Regarding intergenic spacers, *N. nyemitawus* possesses 12 spacers totaling 183 bp (range 1 to 60 bp), while *N. viridimaculatus* has 15 spacers totaling 366 bp (range 1 to 237 bp).

**Table 2.** Gene organization of the mitochondrial genomes of *N. nyemitawus* and *N. viridimaculatus*. Slash separates *N. nyemitawus* (left) and *N. viridimaculatus* (right) data.

**Gene**	**Position**		**Length**	**Intergenic Nucleotides**	**Direction**	**Anticodon**	**Codon**	
	**From**	**To**					**Start**	**Stop**
*trnY*	1/119	65/185	65/67	0/0	–	GUA		
*trnI*	71/189	138/255	68/67	5/3	–	GAU		
*trnC*	141/56	203/118	63/63	2/0	–	GCA		
*ND2*	221/277	1219/1272	999/996	17/21	+		ATT	TAG
*trnS1*	1218/1271	1274/1330	57/60	–2/–2	–	UCU		
*trnW*	1275/1333	1342/1401	68/69	0/2	+	UCA		
*trnN*	1339/1398	1407/1468	69/71	–4/–4	–	GUU		
*trnM*	1408/1471	1471/1535	64/65	0/2	–	CAU		
*ND3*	1476/1541	1829/1894	354/354	4/5	–		ATA	TAA
*trnG*	1827/1892	1890/1959	64/68	–3/–3	–	UCC		
*COIII*	1900/1973	2676/2776	777/804	9/13	–		ATG	TAA
*ATP6*	2696/2780	3382/3454	687/675	19/3	–		ATG	TAA
*ATP8*	3376/3692	3534/3844	159/153	–7/237	–		ATT/ATC	TAA
*trnD*	3535/3845	3604/3916	70/72	0/0	–	GUC		
*trnK*	3609/3921	3680/3992	72/72	4/4	+	UUU		
*COII*	3681/4005	4361/4685	681/681	0/12	–		ATT	TAA
*trnL2*	4362/4686	4426/4749	65/64	0/0	–	UAA		
*COI*	4429/4752	5961/6284	1533/1533	2/2	–		ATG	TAA
*trnE*	5960/6286	6024/6350	65/65	–2/1	+	UUC		
*trnF*	6024/6356	6087/6419	64/64	–1/5	–	GAA		
*ND5*	6088/6420	7770/8102	1683/1683	0/0	–		ATT	TAA
*trnH*	7771/8103	7835/8175	65/73	0/0	–	GUG		
*ND4*	7835/8175	9172/9512	1338/1338	–1/–1	–		ATG	TAA
*ND4L*	9166/9506	9453/9793	288/288	–7/–7	–		ATT	TAA
*trnT*	9453/9798	9518/9867	66/70	–1/4	+	UGU		
*trnP*	9519/9867	9582/9935	64/69	0/–1	–	UGG		
*ND6*	9636/9988	10145/10488	510/501	53/52	+		ATT	TAA
*CYTB*	10145/10488	11277/11627	1133/1140	–1/–1	+		ATA/ATG	TA/TAA
*trnS2*	11285/11626	11351/11692	67/67	7/–2	+	UGA		
*ND1*	11351/11691	12289/12629	939/939	–1/–2	–		ATA	TAA
*trnL1*	12287/12627	12361/12698	75/72	–3/–3	–	UAG		
*lrRNA*	12362/12699	13669/13997	1308/1299	0/0	–			
*trnA*	13670/13998	13732/14063	63/66	0/0	–	UGC		
*srRNA*	13733/14064	14478/14806	746/743	0/0	–			
*trnV*	14479/14807	14545/14870	67/64	0/0	–	UAC		
*trnQ*	14547/14870	14611/14935	65/66	1/–1	–	UUG		
*trnR*	14672/1	14728/55	57/55	60/0	+/–	UCG		
CR	14729/14936	15164/15297	436/362	0/0				

The nucleotide composition of the mitogenome from *N. nyemitawus* was biased toward A and T, with 85.5% A+T content (A = 44.1%, T = 41.4%, C = 8.0%, G = 6.5%). The A+T content was 84.3% and 88.6% in the protein-coding genes and rRNA genes, respectively. The nucleotide composition of the mitogenome of *N. viridimaculatus* was biased toward A and T, with 85.0% A+T content (A = 43.9%, T = 41.1%, C = 8.3%, G = 6.8%). The A+T content was 83.8% and 88.2% in the protein-coding genes and rRNA genes, respectively. The values of AT-skew and GC-skew are often used to indicate the nucleotide composition of the mitochondrial genome. In this study, the nucleotide features of the two new mitogenomes were investigated by calculating the AT-skew and GC-skew (Table 3). The skew analysis shows that the mitogenome of *N. nyemitawus* had a positive AT-skew (0.032) and a negative GC-skew (–0.103), and the mitogenome of *N. viridimaculatus* had a positive AT-skew (0.033) and a negative GC-skew (–0.099).

**Table 3.** Nucleotide features of the mitochondrial genome across *N. nyemitawus* and *N. viridimaculatus*. Slash separates *N. nyemitawus* (left) and *N. viridimaculatus* (right) data.

**Feature**	**Length (bp)**	**T%**	**C%**	**A%**	**G%**	**A+T%**	**AT-Skew**	**GC-Skew**
Whole genome	15164/15297	41.4/41.1	8.0/8.3	44.1/43.9	6.5/6.8	85.5/85.0	0.032/0.033	–0.103/–0.099
*ATP6*	687/675	38.4/37.0	8.4/9.5	44.3/44.7	8.9/8.7	82.7/81.7	0.071/0.094	0.029/–0.044
*ATP8*	159/153	44.7/40.5	1.9/2.6	49.1/49.7	4.4/7.2	93.8/90.2	0.047/0.102	0.397/0.469
*CO1*	1533/1533	34.0/33.1	11.9/12.4	43.2/43.6	10.8/10.8	77.2/76.7	0.119/0.137	–0.048/–0.069
*CO2*	681/681	39.1/36.4	9.7/9.8	43.0/44.5	8.2/9.3	82.1/80.9	0.048/0.100	–0.084/–0.026
*CO3*	777/804	33.8/34.8	11.7/10.2	45.4/45.8	9.0/9.2	79.2/80.6	0.146/0.136	–0.130/–0.052
*CYTB*	1133/1140	46.0/45.8	9.7/10.2	34.2/34.2	10.2/9.8	80.2/80.0	–0.147/–0.145	0.025/–0.020
*ND1*	939/939	37.8/39.1	8.9/9.1	46.5/44.8	6.7/7.0	84.3/83.9	0.103/0.068	–0.141/–0.130
*ND2*	999/996	49.7/50.8	4.0/4.8	42.6/40.2	3.6/4.2	92.4/91.0	–0.077/–0.116	–0.053/–0.067
*ND3*	354/354	38.4/37.0	5.4/8.8	49.4/48.0	6.8/6.2	87.8/85.0	0.125/0.129	0.115/–0.173
*ND4*	1338/1338	38.1/37.7	7.8/7.8	48.0/48.2	6.1/6.3	86.1/85.9	0.115/0.122	–0.122/–0.106
*ND4L*	288/288	39.9/40.3	8.7/9.4	50.3/48.3	1.0/2.1	90.2/88.6	0.115/0.090	–0.794/–0.635
*ND5*	1683/1683	38.7/39.2	7.4/7.1	47.7/47.4	6.2/6.4	86.4/86.6	0.104/0.095	–0.088/–0.052
*ND6*	510/501	47.8/48.1	4.5/5.4	43.9/42.5	3.7/4.0	91.8/90.6	–0.043/–0.062	–0.095/–0.149
*srRNA*	746/743	46.9/48.9	7.4/7.0	41.7/39.4	4.0/4.7	88.6/88.3	–0.059/–0.108	–0.298/–0.197
*lrRNA*	1308/1299	45.0/44.5	7.1/7.1	43.6/43.6	4.4/4.8	88.6/88.1	–0.016/–0.010	–0.235/–0.193
CR	436/362	44.7/45.9	7.3/4.1	43.6/46.4	4.4/3.6	88.3/92.3	–0.012/0.005	–0.248/–0.065
*PCGs*	11081/11085	39.8/39.7	8.4/8.7	44.5/44.1	7.3/7.5	84.3/83.8	0.056/0.053	–0.070/–0.074
*rRNAs*	2054/2042	45.7/46.1	7.2/7.1	42.9/42.1	4.2/4.8	88.6/88.2	–0.032/–0.045	–0.263/–0.193
*tRNAs*	1443/1469	45.0/45.5	6.9/6.6	43.4/43.5	4.8/4.4	88.4/89.0	–0.018/–0.022	–0.179/–0.200

### Protein-coding genes and codon usage

In the mitochondrial genomes of *N. nyemitawus* and *N. viridimaculatus*, the total lengths of the protein-coding genes (PCGs) are 11,081 bp and 11,085 bp, accounting for 73.07% and 72.47% of the full genome, respectively. The PCGs of both species exhibit a pronounced AT-rich feature, with A+T contents of 84.3% and 83.8%, respectively. The A+T content varies considerably among individual genes, with the lowest value of 76.7% (the *COI* gene of *N. viridimaculatus*) and the highest value of 93.8% (the *ATP8* gene of *N. nyemitawus*).

**Figure 2.**
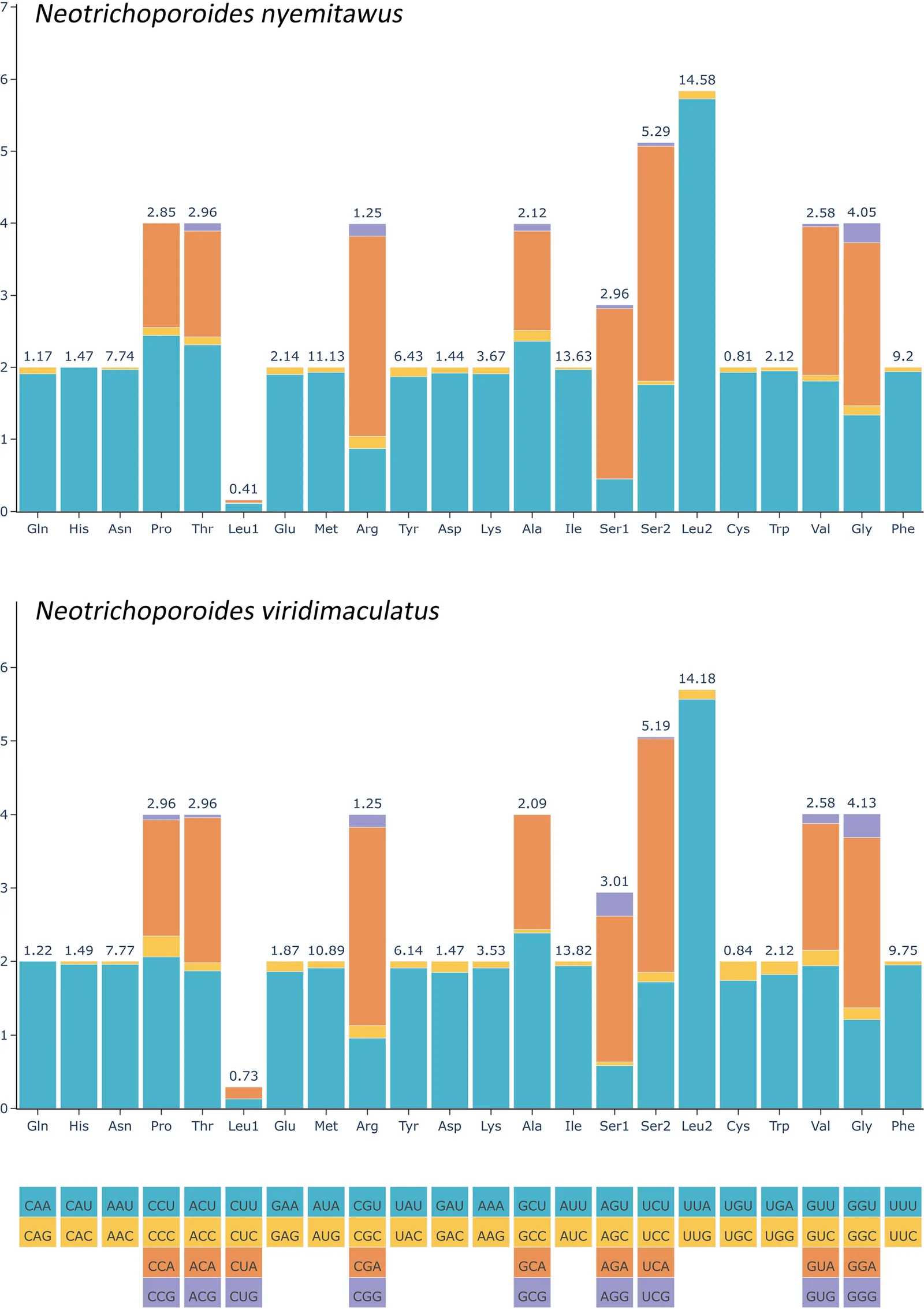
Relative synonymous codon usage in mitochondrial genomes of *N. nyemitawus* and *N. viridimaculatus*. The X-axis represents codon families, and the Y-axis shows RSCU values. Cyan and orange layers indicate codons with A/U (A/T-ending) at the third position, whereas yellow and purple layers indicate codons with G/C (G/C-ending) at the third position.

Calculation of the relative synonymous codon usage (RSCU) was undertaken to further characterize the high A+T content and the associated codon usage profile of the mitogenome (Fig. 2). The results showed that both species significantly preferred codons ending with A or U. Specifically, codons with RSCU values greater than 2 in *N. nyemitawus* included UUA (Leu2), GUA (Val), and CGA (Arg); whereas in *N. viridimaculatus*, they included UUA (Leu2), GUA (Val), GAA (Glu), and CCU (Pro), all of which exhibited extremely high usage frequencies.

**Figure 3.**
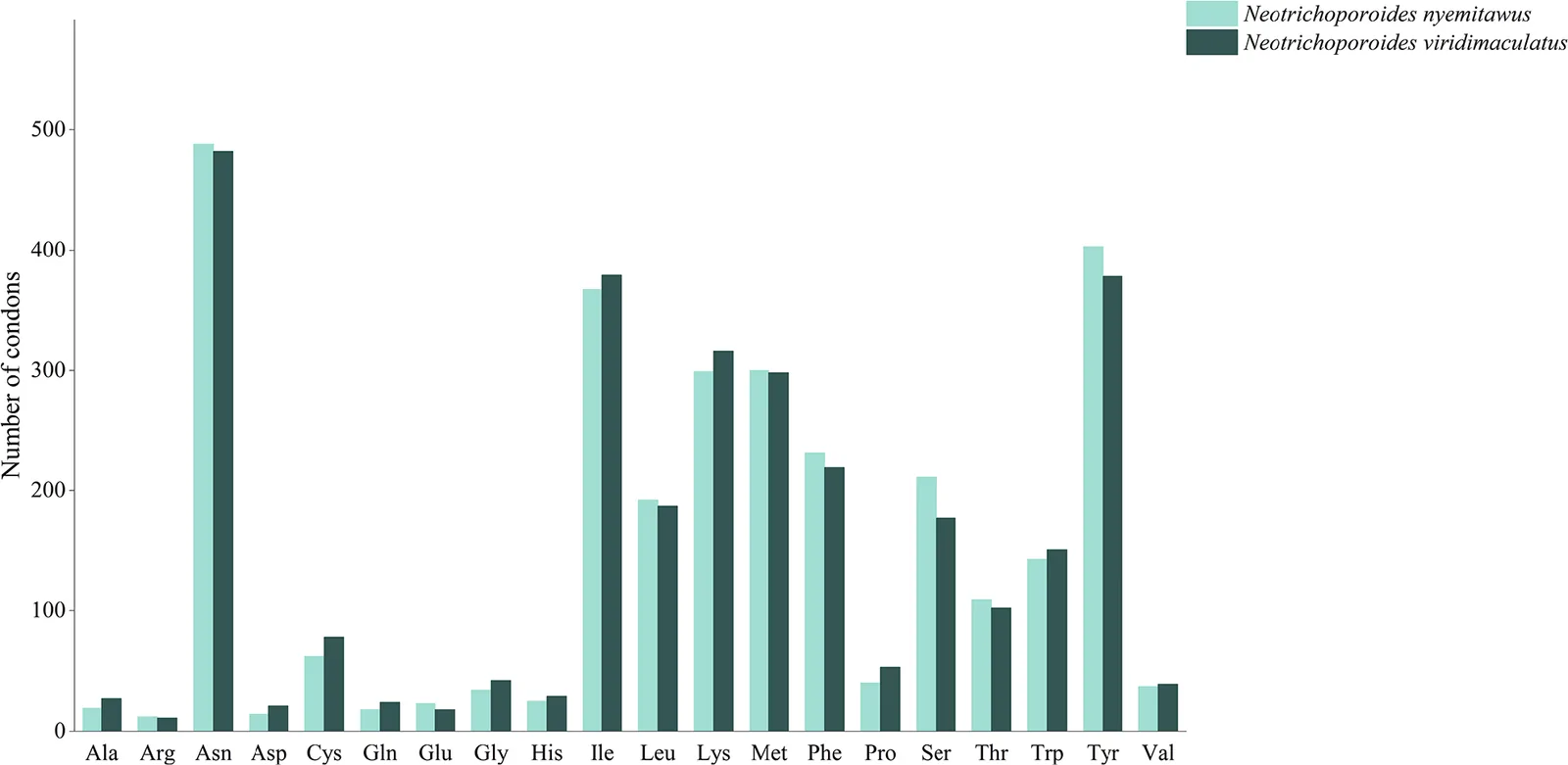
Codon distribution in the mitochondrial genomes of *N. nyemitawus* and *N. viridimaculatus*. Numbers on the Y-axis indicate the total number of codons, and codon families are shown on the X-axis. Different colors represent different species.

Codon distribution analysis showed that, in both genomes, the most frequently used codons were AAU (Asn), UAU (Tyr), AUU (Ile), and AAA (Lys), whereas codons ending with G or C, including CUG, CAC, CAG, and GAG, were the least frequent (Fig. 3). Codons ending in A or U overwhelmingly predominate in absolute numbers, shaping the overall codon usage pattern, which is one of the major reasons for the observed A+T bias in these mitogenomes.

The two *Neotrichoporoides* species share similar start- and stop-codon patterns. All protein-coding genes use ATN (ATA, ATG, ATT, ATC) as start codons. Stop codons include TAA, TAG, and truncated stop codons (e.g., TA for *CYTB* in *N. nyemitawus*), with TAA being the most frequently used. Truncated stop codons are widespread in metazoan mitochondrial genomes and are thought to be completed via post-transcriptional polyadenylation (Ojala et al. 1981).

**Figure 4.**
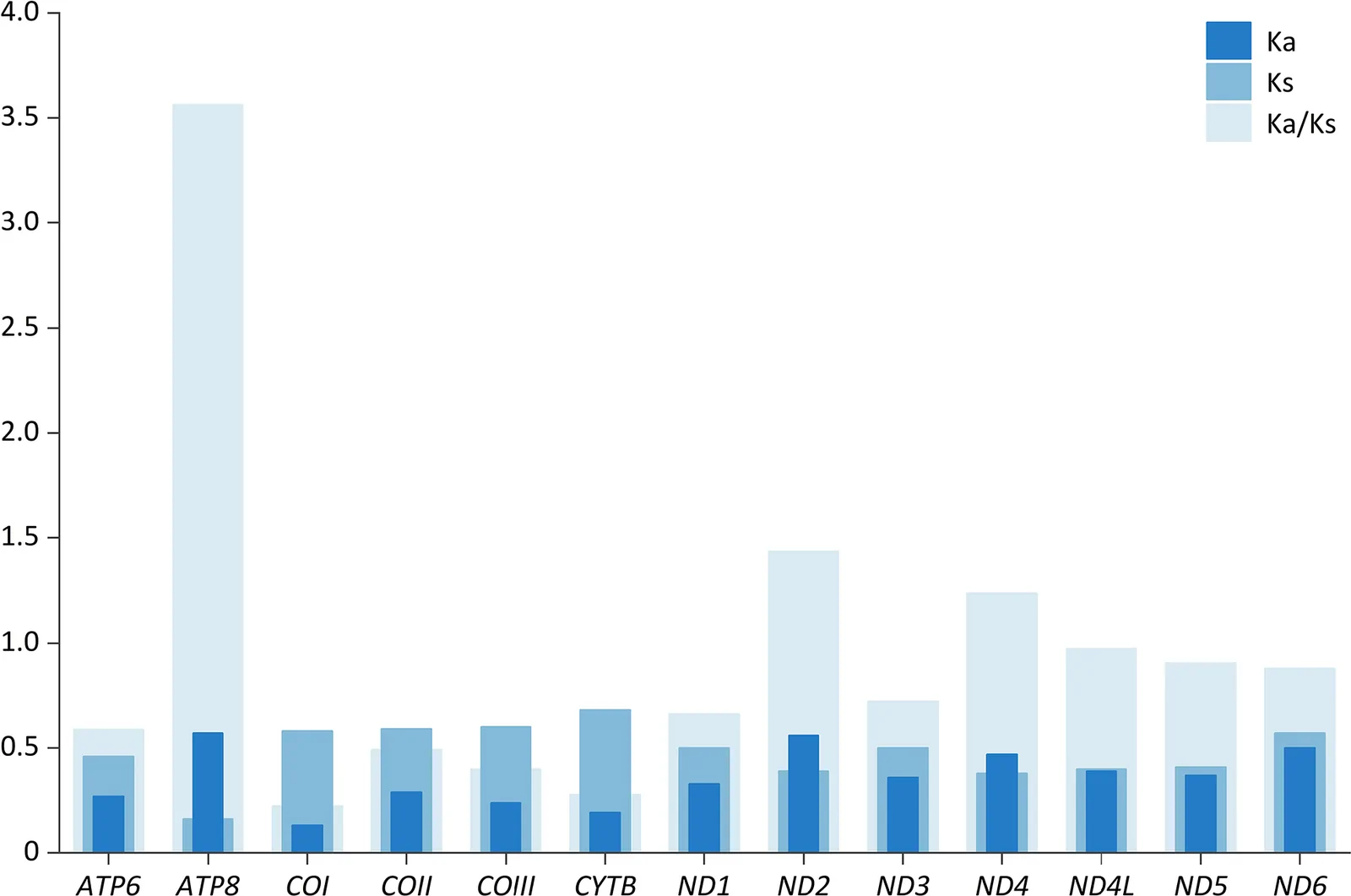
Evolutionary rates of 13 PCGs in Eulophidae mitogenomes. The X-axis and Y-axis indicate PCGs and Ka/Ks ratios (nonsynonymous/synonymous substitution rates), respectively. Ka, Ks, and Ka/Ks are color-coded as indicated in the legend.

Based on nine mitochondrial genomes of Eulophidae, with *Exoristobia philippinensis* as the outgroup, we calculated the nonsynonymous substitution rate (Ka), synonymous substitution rate (Ks), and Ka/Ks ratios for the 13 protein-coding genes (Fig. 4). Among the 13 genes, *ATP8* and *ND2* exhibited the highest Ka and Ka/Ks ratios, indicating that these two genes have the highest variability. The evolutionary rate ranking of the 13 genes was as follows: *ATP8* > *ND2* > *ND4* > *ND4L* > *ND5* > *ND6* > *ND3* > *ND1* > *ATP6* > *COII* > *COIII* > *CYTB* > *COI.*

Notably, the Ka/Ks ratios of *ATP8*, *ND2*, and *ND4* all exceeded 1, which may be suggestive of positive selection. The Ka/Ks ratio of *ND4L* was 0.98, close to the neutral expectation of 1, suggesting that this gene may not be under strong selective pressure. Among the remaining genes, *ND5* and *ND6* had ratios of 0.90 and 0.88, respectively, slightly below 1, suggesting only weak purifying selection, whereas the other genes had ratios far below 1, indicating strong purifying selection.

High Ka/Ks ratios for *ATP8* have been reported in other species (Jia et al. 2020; Xu et al. 2021). The rapid evolution of most genes may be associated with differences in ecology, host range, and reproductive strategies among eulophid species (Kang and Hu 2023), potentially reflecting adaptation to diverse environments. However, such rate acceleration could also result from locus-specific variation in evolutionary constraints, and further functional or population-level studies are needed to confirm selective pressures.

### Transfer RNA genes, ribosomal RNA genes, and control region

The mitochondrial genomes of both species encode the same number of tRNA genes (22 in each), yet their total lengths differ by 27 bp—1443 bp in *N. nyemitawus* versus 1469 bp in *N. viridimaculatus*—with individual gene sizes ranging from 55 to 75 bp. In terms of copy number, *trnL* and *trnS* each possess two copies, whereas the remaining 20 tRNAs occur as single copies. The anticodon complement is highly consistent with that reported for other known hymenopteran species, showing no deviation from the general pattern observed in this order. At the secondary structural level, the majority of tRNAs adopt the canonical cloverleaf folding; however, *trnS1* and *trnR* represent structurally degenerate forms, as both lack the DHU arm and are consequently folded into a simple loop configuration. Notably, this simplification in *trnS1* is not an incidental feature but a recurrent architectural motif in insect mitochondrial genomes (Dowton et al. 2002). Length variation among tRNA sequences is largely attributable to differences in the DHU and TΨC arms (Shao et al. 2001). Additionally, the anticodon of *trnS1* is UCU, rather than the more frequently encountered GCU in insect mitochondria.

In *N. nyemitawus*, a total of 14 mismatched base pairs were found in the tRNA arm structures. Apart from typical Watson-Crick pairings (A-U, G-C), G-U pairs (non-canonical/wobble pairings) were present in *trnI*, *trnC*, *trnW*, *trnN*, *trnG*, *trnD*, *trnF*, *trnT*, *trnP*, *trnA*, *trnQ*, *and trnR*, and a U-U mismatch was found in *trnV*. In *N. viridimaculatus*, 11 mismatched base pairs were detected in the tRNA arm structures. G-U pairs occurred in *trnC*, *trnM*, *trnG*, *trnD*, *trnF*, *trnT*, *trnP*, *trnA*, and *trnQ* (with three G-U in *trnA*).

In both *Neotrichoporoides* species, the *lrRNA* and *srRNA* genes are encoded on the antisense strand and exhibit a pronounced A+T bias. The *lrRNA* and *srRNA* genes are located between *trnL1* and *trnA*, and between *trnA* and *trnV*, respectively. In *N. nyemitawus*, the *lrRNA* is 1308 bp in length with an A+T content of 88.6%, and the *srRNA* is 746 bp with an A+T content of 88.6%. In *N. viridimaculatus*, the *lrRNA* is 1,299 bp with an A+T content of 88.1%, and the *srRNA* is 743 bp with an A+T content of 88.3%.

The largest non-coding region in the mitochondrial genome is the A+T-rich region (i.e., the control region), which regulates mitochondrial DNA replication and transcription (Boore 1999; Cameron 2014). In *N. nyemitawus*, the control region is 436 bp in length and located between *trnR* and *trnY*, whereas in *N. viridimaculatus* it is 362 bp and located between *trnQ* and *trnR*. The A+T content of the control region is 88.3% and 92.3% in the two *Neotrichoporoides* species, respectively. AT-skew and GC-skew analysis indicates that the control region of *N. nyemitawus* exhibits a preference for T and C bases, whereas that of *N. viridimaculatus* shows a preference for A and C bases.

### Gene arrangement patterns

In the evolutionary history of insects, mitochondrial genome rearrangements are generally rare events (Cameron 2014). However, within Hymenoptera, such rearrangements are being discovered in an increasing number of species (Shao et al. 2003). Specifically, the gene order remains relatively conserved in Symphyta, whereas rearrangements occur increasingly frequently in Apocrita (Wei et al. 2010). Studies on Chalcidoidea have revealed extensive gene rearrangements within this lineage, involving numerous tRNA genes and some protein-coding genes (Tang et al. 2018). In addition, large-scale rearrangements of protein-coding genes have also been observed in multiple lineages of Hymenoptera (Xiao et al. 2011; Wei et al. 2014).

**Figure 5.**
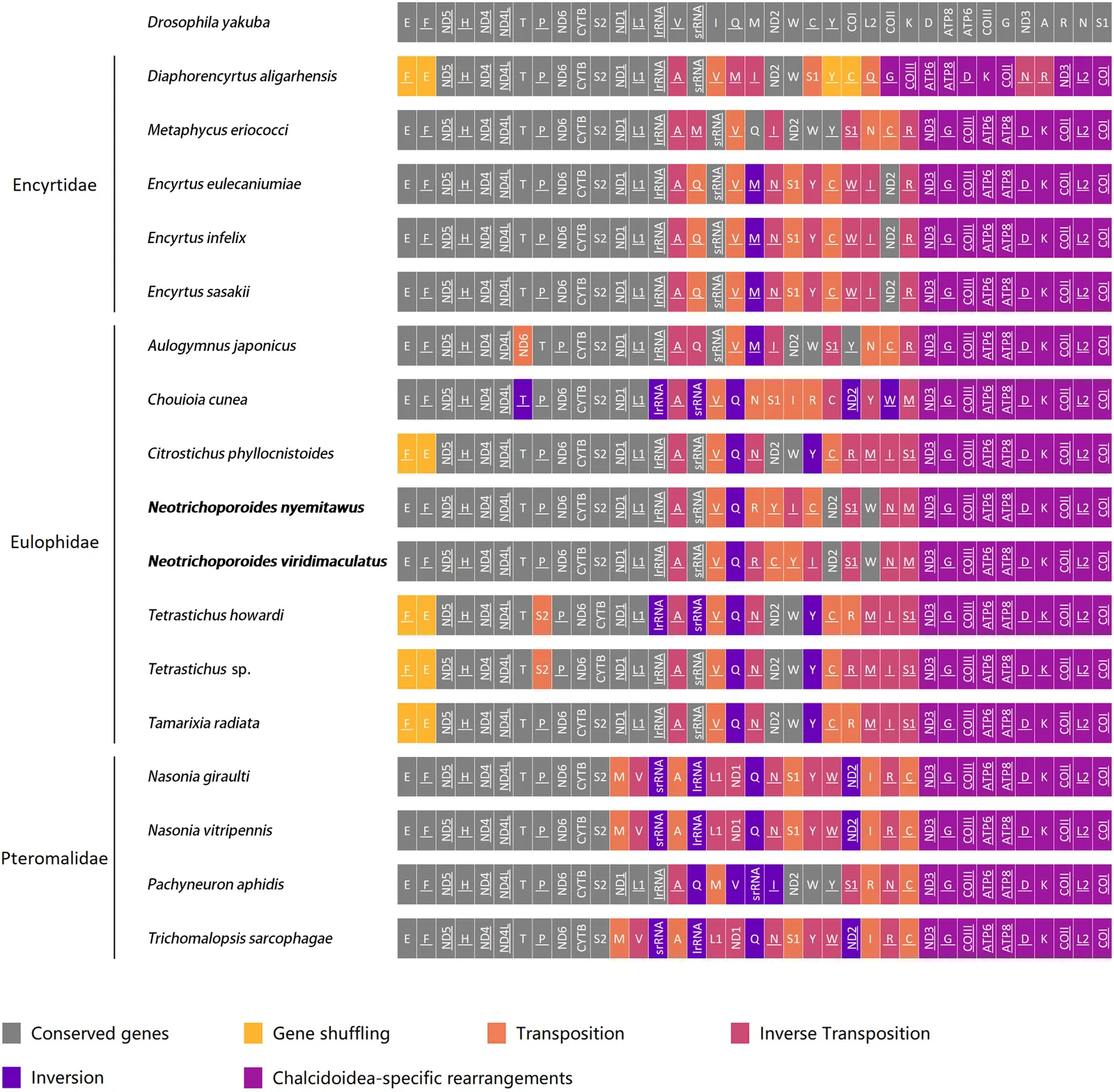
Comparison of mitochondrial gene arrangements among 17 chalcidoid species. The gene order of *Drosophila yakuba* is used as the putative ancestral arrangement. Conserved gene blocks are shown in gray, and rearranged regions are highlighted in colored boxes. Gene names are abbreviated according to standard insect mitochondrial genome annotations; gene names with underlines indicate N-strand coding, while those without indicate J-strand coding.

This study analyzed mitochondrial genome data from 17 species across three families of Chalcidoidea (Encyrtidae, Eulophidae, Pteromalidae), using the gene arrangement of *Drosophila yakuba* as the putative ancestral pattern to examine gene rearrangements (Shao and Barker 2003). The results showed that among the 17 species above, rearrangements occurred mainly on tRNA genes, whereas the 13 protein-coding genes exhibited few rearrangements and their order remained largely conserved (Fig. 5).

In each of the five Encyrtidae species, *trnA* and *trnR* underwent inverse transposition, and *trnV* underwent transposition.

In the eight species of Eulphidae, the protein-coding genes were relatively conserved, with only *ND6* of *Aulogymnus japonicus* undergoing transposition. Among the rRNA genes, only *srRNA* and *lrRNA* of *Chouioia cunea* and *Tetrastichus howardi* underwent inversion. In addition, *tr*n*A* underwent inverse transposition and *trnV* underwent transposition in all eight species, a finding reported for the first time in Eulphidae. The gene arrangements of *N. nyemitawus* and *N. viridimaculatus* are similar, with differences only in *trnR*, *trnY*, *trnI*, and *trnC*: in *N. nyemitawus*, *trnR* is encoded on the J-strand, and the remaining three tRNA genes are arranged as *trnY*–*trnI*–*trnC*; whereas in *N. viridimaculatus*, *trnR* is encoded on the N-strand, and the remaining three tRNA genes are arranged as *trnC*-*trnY*-*trnI*.

In the four species of Pteromalidae, *trnM* and *trnC* all underwent transposition, and *trnN* all underwent inverse transposition. Among the rRNA genes, all except *lrRNA* of *Pachyneuron aphidis* underwent inversion.

Meanwhile, the gene cluster *COI*-*trnL2*-*COII*-*trnK*-*trnD*-*ATP8*-*ATP6*-*COIII*-*trnG*-*ND3* underwent a specific rearrangement, becoming *ND3*-*trnG*-*COIII*-*ATP6*-*ATP8*-*trnD*-*trnK*-*COII*-*trnL2*-*COI.*

### Phylogenetic relationships

**Figure 6.**
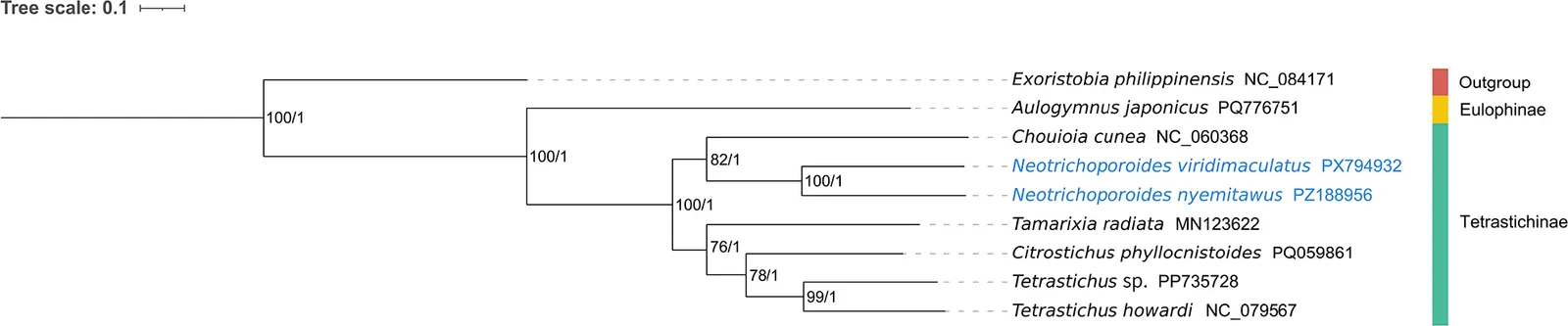
Phylogenetic tree inferred from Bayesian inference (BI) and maximum likelihood (ML) analyses based on the 13 protein-coding genes from eight Eulophidae species and one outgroup from Encyrtidae. Nodal support values are shown as BI posterior probabilities (right) and ML bootstrap values (left). Red indicates the outgroup (Encyrtidae), yellow represents Eulophinae, and green represents Tetrastichinae. Species names in blue indicate the two newly sequenced species in this study.

The results of ML and BI phylogenetic analyses based on the concatenated dataset are shown in Fig. 6. Using *Exoristobia philippinensis* as an outgroup, and combining data from the NCBI database with the two newly added mitochondrial genomes of *Neotrichoporoides* species, a phylogenetic tree of Encyrtidae was constructed from 13 protein-coding gene sequences across nine mitochondrial genomes.

The maximum likelihood (ML) and Bayesian inference (BI) analyses produced congruent results. The resulting phylogenetic tree shows the following branching relationships: *Aulogymnus japonicus* + ((*Chouioia cunea* + (*Neotrichoporoides nyemitawus* + *Neotrichoporoides viridimaculatus*)) + (*Tamarixia radiata* + (*Citrostichus phyllocnistoides* + (*Tetrastichus* sp. + *Tetrastichus howardi*)))).

Overall, the phylogenetic trees constructed using both methods show that congeneric species form well-supported monophyletic clades (*N. nyemitawus* and *N. viridimaculatus* cluster together, as do *Tetrastichus* sp. and *Tetrastichus howardi*), which is consistent with the morphological classification. At the subfamily level, morphological classification divides Eulophidae into Entedoninae, Entiinae, Eulophinae, Tetrastichinae, and Opheliminae, among which *Aulogymnus japonicus* belongs to Eulophinae, whereas the remaining species belong to Tetrastichinae. Species from these two subfamilies together form a monophyletic group. In the phylogenetic trees reconstructed using maximum likelihood and Bayesian inference, the two subfamilies formed distinct clades, with the divergence node positioned near the base of the tree and receiving strong support values. Furthermore, because this concatenated dataset includes all three codon positions of protein-coding genes, it provides richer information on molecular variation, which may be useful for phylogenetic studies of Eulophidae, particularly for species with problematic or unusual taxonomic placements.

## Conclusions

In this study, we report the first mitochondrial genomes of two *Neotrichoporoides* species, *N. nyemitawus* and *N. viridimaculatus*. The two newly sequenced genomes show high similarity in genome size, nucleotide composition, A+T bias, AT-skew, GC-skew, codon usage of protein-coding genes, tRNA secondary structures, and gene rearrangements.

In Eulophidae, mitochondrial genome rearrangements are mainly concentrated on tRNA genes, such as the inverse transposition of *trnA* and the transposition of *trnV*. Furthermore, the gene arrangements of *N. nyemitawus* and *N. viridimaculatus* are similar, with only minor rearrangement differences in a few tRNA genes.

Phylogenetic analyses of the concatenated dataset using Bayesian inference (BI) and maximum likelihood (ML) produced well-supported phylogenetic trees with clear topological structures, providing a useful analytical framework and high-quality data for future studies aiming to resolve the phylogenetic relationships of Eulophidae. Although this study contributes to clarifying the phylogenetic relationships of some eulophid lineages, it still has limitations, including limited sample size and insufficient molecular evidence. Future research should increase species sampling, expand mitochondrial genome datasets, and integrate multiple types of data to conduct robust phylogenetic analyses that comprehensively evaluate the internal classification of Eulophidae.

## Additional information

### Artificial Intelligence (AI) use

The authors accept full responsibility for the content of the manuscript, including the disclosure of any use of AI.

No AI tools were used in the preparation of this manuscript.

### Author ORCIDs

J.-S. Wen https://orcid.org/0009-0002-7801-2602

X.-X. Jin https://orcid.org/0009-0004-6154-1991

W.-J. Li https://orcid.org/0009-0004-9790-3608

Z.-P. Chen https://orcid.org/0009-0006-9947-5074

G.-H. Zu https://orcid.org/0000-0002-9892-2171

### Data availability

All of the data that support the findings of this study are available in the main text or Supplementary Information.

## Supplementary materials

Supplementary material 1 Phylogenetic trees file of ML Data type tree https://binary.pensoft.net/file/1765133This dataset is made available under the Open Database License (http://opendatacommons.org/licenses/odbl/1.0/). The Open Database License (ODbL) is a license agreement intended to allow users to freely share, modify, and use this Dataset while maintaining this same freedom for others, provided that the original source and author(s) are credited. Jun-Sheng Wen, Xiang-Xiang Jin, Wen-Jian Li, Zhi-Peng Chen, Guo-Hao Zu

Supplementary material 2 Phylogenetic trees file of BI Data type tree https://binary.pensoft.net/file/1765134This dataset is made available under the Open Database License (http://opendatacommons.org/licenses/odbl/1.0/). The Open Database License (ODbL) is a license agreement intended to allow users to freely share, modify, and use this Dataset while maintaining this same freedom for others, provided that the original source and author(s) are credited. Jun-Sheng Wen, Xiang-Xiang Jin, Wen-Jian Li, Zhi-Peng Chen, Guo-Hao Zu
